# SDF-1/CXCR4 Signaling Maintains Stemness Signature in Mouse Neural Stem/Progenitor Cells

**DOI:** 10.1155/2017/2493752

**Published:** 2017-03-20

**Authors:** Shih-Yin Ho, Thai-Yen Ling, Hsing-Yu Lin, Jeffrey Tsai-Jui Liou, Fei-Chih Liu, I-Chun Chen, Sue-Wei Lee, Yu Hsu, Dar-Ming Lai, Horng-Huei Liou

**Affiliations:** ^1^Department of Neurology, National Taiwan University Hospital, College of Medicine, National Taiwan University, Taipei 10051, Taiwan; ^2^Department of Pharmacology, College of Medicine, National Taiwan University, Taipei 10051, Taiwan; ^3^Department of General Internal Medicine, Taipei Medical University Hospital, College of Medicine, Taipei Medical University, Taipei 11042, Taiwan; ^4^Department of Surgery, Division of Neurosurgery, National Taiwan University Hospital, College of Medicine, National Taiwan University, Taipei 10051, Taiwan; ^5^National Taiwan University Hospital, Yunlin Branch, Douliu 64041, Taiwan

## Abstract

SDF-1 and its primary receptor, CXCR4, are highly expressed in the embryonic central nervous system (CNS) and play a crucial role in brain architecture. Loss of SDF-1/CXCR4 signaling causes abnormal development of neural stem/progenitor cells (NSCs/NPCs) in the cerebellum, hippocampus, and cortex. However, the mechanism of SDF-1/CXCR4 axis in NSCs/NPCs regulation remains unknown. In this study, we found that elimination of SDF-1/CXCR4 transduction caused NSCs/NPCs to lose their stemness characteristics and to encounter neurogenic differentiation. Moreover, Notch and RE1 silencing transcription factor (REST) both play an essential role in NSCs/NPCs maintenance and neuronal differentiation and were dramatically downregulated following SDF-1/CXCR4 cascade inhibition. Finally, we demonstrated that the expression of achaete-scute homolog 1 (Ascl1), a proneural gene, and p27, an antiproliferative gene, were significantly increased after genetic elimination of SDF-1 alleles. Our results support that the loss of functional SDF-1/CXCR4 signaling pathway in NSCs/NPCs induces exit of cell cycle and promotes premature neural differentiation.

## 1. Introduction

Neurogenesis is the process of generating functional neurons from NSCs/NPCs in the mammalian brain. Although most neurogenesis occurs during the embryonic period, there are two restricted regions of the brain including subventricular zone (SVZ) and subgranular zone (SGZ) that can renew and give rise to new neurons throughout the lifespan of individuals [1]. It has been known that NSCs/NPCs has highly dynamic interaction with their niches to govern self-renewal or differentiation [2]. Extensive studies have demonstrated that neurogenesis is affected by a variety of physiological or pathological stimuli and plays a vital role in learning function and brain repair after injury [3–5]. Therefore, it is important to identify the key signaling cascade responsible for NSCs/NPCs regulation.

Chemokines are low molecular weight proteins that are classified into four subfamilies, CXC, CC, C, and CX3C, based on conserved cysteine residues [6] and are responsible for varied cellular functions, such as directing leukocyte migration, regulating T cell differentiation, and angiogenesis [7, 8]. In addition to their physiological roles in immune system, chemokines also contribute to maintaining normal brain morphogenesis during the embryonic stages [9, 10]. For example, stromal cell-derived factor 1 (SDF-1), also known as CXCL12, and its primary receptor, CXCR4, are highly expressed in the CNS [11–13]. Genetic deletion of the gene for the SDF-1 or CXCR4 results in embryonic lethality [14, 15]. Moreover, previous studies reveal that mice deficient in either SDF-1 or CXCR4 receptors show abnormal development of the cerebellum, hippocampal dentate gyrus (DG), and cortex in the embryonic brain as a result of ectopic positioning of neuronal precursor cells [14, 15].

Histological staining from mouse brain sections indicates high level of CXCR4 expression in SGZ and SVZ implicating that the SDF-1/CXCR4 signaling pathway may be involved in NSCs/NPCs regulation [16–18]. As mentioned above, in vitro studies from human or murine NPCs cultures showed that the SDF-1/CXCR4-mediated pathway played an important role in NPCs migration or proliferation [17, 19–21]. However, the roles of SDF-1/CXCR4 on NSCs/NPCs regulation and its associated signaling mechanisms remain unknown.

In the present study, we examined the potential functions of the SDF-1/CXCR4-triggered signaling pathway in maintenance of NSCs/NPCs using immunocytochemical, flow cytometric, and genetic engineering techniques. Our results show that the SDF-1/CXCR4 cascade may play an unreported role in regulating “stemness” characteristics or neuronal lineage differentiation in NSCs/NPCs.

## 2. Material and Methods

### 2.1. Animals

Wild-type C57BL/6 mice were obtained from the Animal Center of National Taiwan University (Taipei, Taiwan), and SDF-1 transgenic mice [22], SDF-1^F/+^;TgCAGGCreER (abbreviated as SDF-1^F/+^;Tg) or SDF-1^F/−^; TgCAGGCreER (SDF-1^F/−^;Tg), were kindly provided by Dr. Dar-Ming Lai (National Taiwan University Hospital). All of the animal experiments were performed in accordance with guidelines established by the Institutional Animal Care and Use Committee of the National Taiwan University College of Medicine.

### 2.2. Neurosphere Culture

Neurosphere cultures were prepared as described by Imura et al. [23]. Briefly, mouse pups of age postnatal day 1 were sacrificed by decapitation and the brains were quickly removed into chilled HEPES solution and dissected by surgical blade. Dissected brains were incubated with digestion buffer and then neutralized with 10% heat-inactivated fetal bovine serum (Invitrogen). Cells were dissociated by gentle mechanical pipetting and resuspended at 2 × 10^5^ cells/mL in serum-free DMEM/F12 medium (Gibco-BRL), which is supplemented with N2 (Gibco-BRL), basic fibroblast growth factor (bFGF, 20 *μ*g/mL; Sigma-Aldrich), and epidermal growth factor (EGF, 10 *μ*g/mL; Sigma-Aldrich). Cells were incubated at 37°C with 5% CO_2_ for 6 days to obtain the primary neurospheres and growth factors were added every 3 days. In the AMD-treated group, AMD were dissolved in culture medium and added every 3 days. To evaluate differentiation ability, 6 days in vitro (DIV) neurospheres were plated onto 0.1% fibronectin- (Sigma-Aldrich) coated dishes in bFGF/EGF-free neurobasal medium supplemented with N2 for 4 days.

### 2.3. Immunocytochemistry

Neurospheres were attached to a glass slide by using a cytocentrifuge (Cyto-Tek) at 1000 rpm for 3 minutes. Cells were fixed with acetone : methanol 1 : 1 at 4°C, washed with PBS, and treated with 3% BSA for at least 1 hour at room temperature. Cells were then incubated with the following primary antibodies at 4°C overnight: rabbit anti-Ki67 (1 : 1000, Abcam), mouse anti-nestin (1 : 300, Abcam), goat anti-CXCR4 (1 : 300, Abcam), rabbit anti-*β*III tubulin (1 : 300, Epitomics), rabbit anti-GFAP (1 : 7000, Abcam), rabbit anti-SOX2 (1 : 300, Abcam), and rabbit anti-CNPase (1 : 1000. Abcam). Secondary antibodies (goat anti-rabbit, goat anti-mouse, and goat anti-rat) were utilized conjugated with Alexa Fluor 594 (1 : 1000, Abcam). Images were taken with confocal laser scanning microscope (LSM780, Carl Zeiss).

### 2.4. Fluorescence-Activated Cell Sorting (FACS)

For FACS analysis, the neurospheres were dissociated into single cell by accutase digestion. Single cell suspensions were then stained with appropriate antibodies as follows: mouse anti-nestin (1 : 50, Abcam), rat anti-CXCR4 (1 : 50, BioLegend), and rabbit anti-*β*III tubulin (1 : 10, Epitomics). All flow cytometric analysis was performed using FACSCAria II (BD Biosciences) and results were analyzed by FACSDiva software (BD Biosciences).

### 2.5. Western Blot Assay

Proteins in cell culture homogenates were separated by 12% SDS-PAGE and transferred onto PVDF membranes (Amersham). Primary antibodies used in immunoblot experiments were as follows: rabbit anti-SOX2 (1 : 10000, Genetex), rabbit anti-*β*III tubulin (1 : 3000, Epitomics), rabbit anti-Ascl (1 : 1000, Abcam), rabbit anti-Notch (1 : 1000, Abcam), mouse anti-p27 (1 : 6000, BD Biosciences), rabbit anti-*α*-tubulin (1 : 150000, Abcam), and rabbit anti-actin (1 : 15000, Epitomics). After incubation with the primary antibodies, horseradish peroxidase-conjugated secondary antibodies were incubated to the blots at a dilution of 1 : 5000–1 : 50000 for 1 hour at room temperature. Chemiluminescence signals were detected using WesternBrightTM ECL (Advansta) and captured with Hyperfilm ECL (Amersham). Immunoblot images were quantitated using ImageJ software (NIH).

### 2.6. Enzyme-Linked Immunosorbent Assay (ELISA)

The quantity of SDF-1 in culture medium was determined as previously described [22]. The ELISA kit for SDF-1 was purchased from R&D Systems, and the manufacturer's protocol was followed.

### 2.7. Reverse Transcription Polymerase Chain Reaction

Total RNA was extracted from secondary neurospheres by using RNeasy Mini Kit (QIAGEN). The cDNA encoding SDF-1 and GAPDH was synthesized by reverse transcription and amplified by Thermal Cycler (Applied Biosystems). The conditions were as follows: 94°C for 2 min before first cycle, denaturation at 95°C for 1 min, annealing at 64°C for 1 min, and extension at 72°C for 2 min followed by 40 cycles and finally 72°C for 10 min after the last cycle. Primer sequences were as follows:

  SDF-1 forward: 5′-AGCCTGAGCTACCGATGCCCCT-3′
  SDF-1 reverse: 5′-GGATGTCAGCCTTCCTCGGGG-3′
  GAPDH forward: 5′-ACCACAGTCCATGCCATCAC-3′
  GAPDH reverse: 5′-TCCACCACCCTGTTGCTGGA-3′

### 2.8. 4-Hydroxytamoxifen Treatment

**Figure 1:**
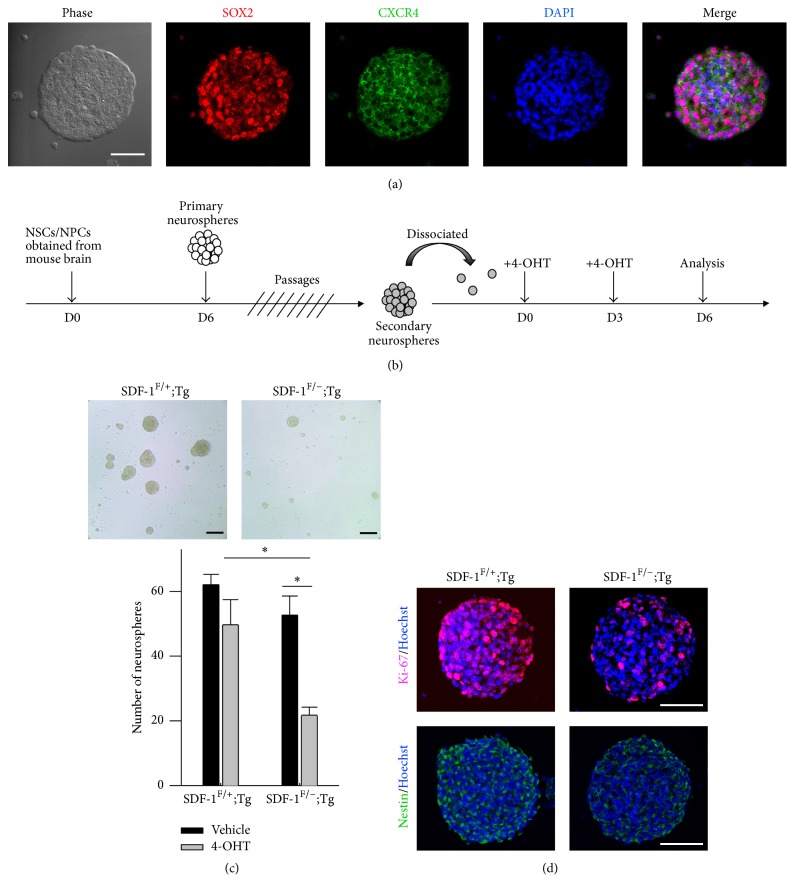
Genetic deletion of SDF-1 gene impairs NSCs/NPCs maintenance. (a) Immunocytochemical staining showed that most cells in neurospheres were SOX2^+^ and coexpressed CXCR4 receptors. Scale bar, 50 *μ*m. (b) Schematic representation of the protocol used in this study. (c) Representative images of secondary neurospheres formation after 4-OHT treatment. SDF-1^F/−^;Tg group showed significant decline in neurospheres outgrowth compared to SDF-1^F/+^;Tg group (upper panel). Scale bar, 100 *μ*m. Quantification of sphere numbers in SDF-1^F/+^;Tg and SDF-1^F/−^;Tg group after vehicle or 4-OHT application (lower panel). Results are representative of three independent experiments with NSCs/NPCs from three independent litters. Data represent the mean ± SEM, ^*∗*^*p* < 0.05, Student's *t*-test. (d) Immunocytochemistrical staining of Ki-67 (red, upper panel) and nestin (green, lower panel) in neurospheres after 4-OHT-induced deletion of SDF-1. Scale bar, 50 *μ*m.

The primary neurospheres isolated from SDF-1^F/+^;Tg or SDF-1^F/−^;Tg mice were as described above. For obtaining a high number of cells for analysis, we performed secondary neurospheres assay in this experiment (Figure 1(b)). Briefly, primary neurospheres were dissociated and expanded in culture for several passages. After the last passage, secondary neurospheres were dissociated into single cells and 4-hydroxytamoxifen (4-OHT, Sigma-Aldrich) was added twice (1DIV and 3DIV) during the 7-day culture period. The neurospheres were harvested and analyzed for deletion efficiency of SDF-1 by RT-PCR.

### 2.9. Tamoxifen Administration

Tamoxifen (Sigma-Aldrich) was dissolved in filter-sterilized corn oil and administered intraperitoneally (150 *μ*g/g body weight) to 4-week-old SDF-1^F/+^;Tg or SDF-1^F/−^;Tg mice in 1-day intervals for 5 consecutive days. Brains were harvested at 1 month after the last injection for immunohistochemical staining.

### 2.10. Morphometric Analysis

The neurospheres obtained from control or AMD-treated groups were dissociated and reseeded into fibronectin-coated dishes in mitogen-free medium allowed to differentiate. After 4 days of differentiation, the neurons were fixed and stained by *β*III tubulin antibody and captured by inverted confocal microscopy. Seven to nine confocal microscopic images were randomly chosen per cultured dish. The average length and branches level of neurite in *β*III tubulin-positive cells were performed with Neurolucida software (MBF Bioscience).

### 2.11. Statistics

Each experiment (except in vivo studies) was independently performed at least three times with similar results. Data were analyzed in SigmaPlot 10.0 software (Systat Software) and expressed as mean ± SEM in each bar graph. The data was evaluated statistically by Student's *t*-test. Statistical significance was set at *p* < 0.05.

## 3. Results

### 3.1. Loss of SDF-1/CXCR4 Pathway Impairs NSCs/NPCs Maintenance

NSCs/NPCs isolated from either embryonic or adult mouse brain can be induced to proliferate in vitro and form a spherical cultures named “neurospheres” when specific mitogens such as bFGF or EGF are provided. These neurospheres are extremely proliferative and expand their size during the culture period and still maintain multipotential ability to differentiate to neurons, astrocytes, and oligodendrocytes [24, 25] (Supplementary Figures  1A-B in Supplementary Material available online at https://doi.org/10.1155/2017/2493752). SDF-1 and its primary receptors, CXCR4, have been reported as being highly expressed in mouse CNS during embryonic stages. Therefore, we first examined the expression of CXCR4 on neurospheres by using immunocytochemical staining. Consistent with previous investigations in human or rat cortical NPCs [21, 26], our results show that CXCR4 was highly colocalized with SOX2-positive (SOX2^+^) cells on neurospheres (Figure 1(a)). Previous studies indicate that mice deficient in CXCR4 or SDF-1 show significant morphological changes in hippocampal DG, sites with continuous genesis and turnover of neurons throughout life. This suggests that SDF-1/CXCR4 signaling pathway may play an important role in neurogenesis. Moreover, blockage of SDF-1/CXCR4 signaling abolished NPCs proliferation under in vitro conditions. However, it is still controversial whether the SDF-1 modulates the biological characteristics of NSCs/NPCs. To test this hypothesis, we used a conditional gene deletion strategy to demonstrate that the loss of SDF-1 would affect the homeostasis of NSCs/NPCs. Primary NSCs/NPCs were isolated from SDF-1^F/+^;Tg and SDF-1^F/−^;Tg mice as described in Materials and Methods. In order to get enough cells for analysis, the secondary neurospheres prepared by multiple passages were used in this study. Twenty-four hours after seeding, cells were treated with 4-OHT for a period before being collected. The floating-spheres were then used for examination (Figure 1(b)). Both RT-PCR amplification and ELISA were used to measure the deletion efficiency. We found that the level of SDF-1 transcripts declined noticeably after 4-OHT applications (Supplementary Figure  2). In addition, the culture media obtained from SDF-1^F/−^;Tg group show a significantly lower amount of SDF-1 than that seen in SDF-1^F/+^;Tg group (SDF-1^F/+^;Tg, 1.8 pg/mL; SDF-1^F/−^;Tg, not detectable). Based on the results above, we next assessed the loss of SDF-1/CXCR4 signaling cascade on neurospheres formation. In cultures treated with 4-OHT, we consistently found a predominant reduction of total neurospheres number (Figure 1(c)). Additionally, in order to further confirm that the SDF-1/CXCR4 pathway is involved in modulating the proliferative capacity in primary neurospheres, we evaluated the DNA replication by Ki-67 staining technique. Interestingly, we found a smaller percentage of the Ki-67-positive (Ki-67^+^) or nestin-positive (nestin^+^) cells in SDF-1^F/−^;Tg group (Figure 1(d)). These results indicate that the SDF-1/CXCR4 pathway may be involved in NSCs/NPCs maintenance in vitro.

To better understand the nature of SDF-1/CXCR4 signaling related to NSCs/NPCs regulation, we utilized the tamoxifen-inducible Cre/loxP system to conditionally delete the SDF-1 gene in adolescent mice and evaluate the impact on hippocampal neurogenesis. Four-week-old SDF-1^F/+^;Tg or SDF-1^F/−^;Tg mice were injected intraperitoneally once daily with tamoxifen for 5 consecutive days, and brains were collected 1 month after the last injection. Our immunohistochemical staining results consistently show a clear decreasing trend in Ki-67^+^, SOX2^+^, and Ki-67^+^/SOX2^+^ cells in SDF-1^F/−^;Tg mice DG after tamoxifen treatment. However, we did not observe a statistically significant difference between SDF-1^F/+^;Tg and SDF-1^F/−^;Tg groups (Supplementary Figures  3A-B). To address whether the reduction of NSCs/NPCs self-renewal by the deletion of SDF-1 allele indeed diminished the neurogenic process in hippocampus, we next stained immature neurons identified by their positivity for doublecortin (DCX). Unexpectedly, we found no significant change in DCX expression in DG (Supplementary Figure  4). These results indicate that the role of SDF-1/CXCR4 cascade in NSCs/NPCs regulation in vivo is more complicated than in vitro culture system.

### 3.2. Inhibition of the SDF-1-Mediated Signaling by CXCR4 Specific Antagonists Disrupted Growth Factor-Induced Proliferation in NSCs/NPCs

**Figure 2:**
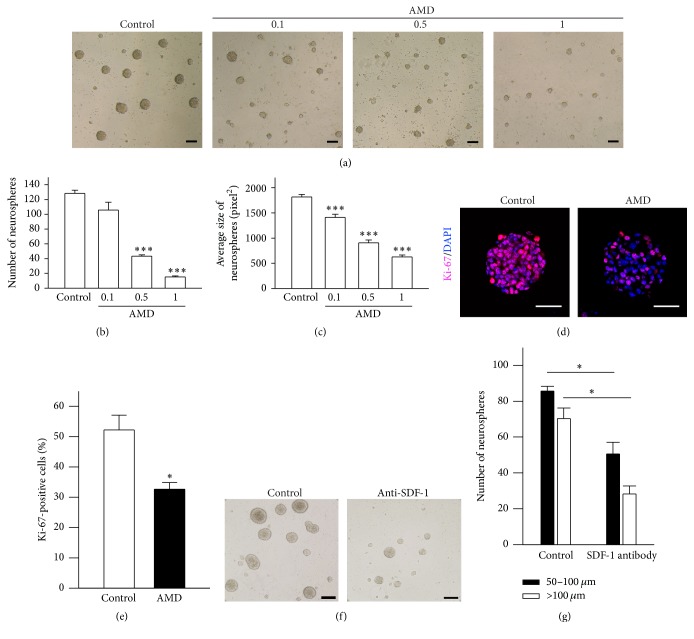
Blocking of SDF-1/CXCR4 pathway impaired growth factor-induced neurospheres formation in vitro. (a) Representative images of 6DIV neurospheres in varying concentration of AMD (0.1, 0.5, and 1.0 *μ*g/mL). Scale bar, 100 *μ*m. (b) (c) CXCR4 specific antagonist, AMD, significantly decreased the number or size of primary neurospheres formation in a dose-dependent manner. Data represent the mean ± SEM, *n* = 3, ^*∗∗∗*^*p* < 0.001. (d) Representative staining images of 6DIV neurospheres. Equal sizes of neurospheres incubated with PBS (control, left panel) or 1.0 *μ*g/mL AMD (AMD, right panel) were stained with cell proliferative marker, Ki-67, to evaluate the self-renewal ability of cells. Scale bar, 50 *μ*m. (e) Quantification of the percentage of Ki-67^+^ cells in a single neurosphere. Data represent the mean ± SEM, *n* = 3, ^*∗*^*p* < 0.05. (f) Representative pictures of neurospheres after 6 days of SDF-1 antibody application. Scale bar, 100 *μ*m. (g) Quantification of neurospheres with medium size (>50 *μ*m) or large size (>100 *μ*m) after SDF-1 antibody neutralization. Data represent the mean ± SEM, *n* = 3, ^*∗*^*p* < 0.05, Student's *t*-test.

To further confirm the observations above, we selectively blocked the SDF-1/CXCR4 signal transduction pathway by CXCR4 receptors-specific antagonists, AMD3100 (AMD), and assessed the outgrowth of cultured mouse NSCs/NPCs. Adding AMD to culture medium on day 1 of primary NSCs/NPCs obtained from mouse brain, we found that AMD significantly decrease the number and/or size of primary neurospheres formation in a dose-dependent manner after 6 days of incubation. Quantification data show that AMD caused about 20%~80% reduction on primary neurospheres outgrowth in both number and size (Figures 2(a)–2(c)) as observed from conditional SDF-1 gene deletion experiments. This inhibitory effect was not due to AMD toxicity on NSCs/NPCs (data not shown). Furthermore, after 6 days of AMD (1 *μ*g/mL) treatment, neurospheres showed obvious decline in Ki-67^+^ cells when compared with the control group (control, 52.3 ± 4.9%; AMD, 32.7 ± 2.2%, Figures 2(d)-2(e)). Previous reports suggest that SDF-1 could directly promote human or rodent NPC proliferation in vitro [21]. However, we did not find a notable increase in number and size of neurospheres when SDF-1 was involved in culture medium (data not shown), consistent with previous findings reported on mice embryonic primary NPCs [19]. These results suggest that there may be a local source of SDF-1 produced by NSCs/NPCs. To investigate the above hypothesis, we neutralized the culture medium SDF-1 with anti-SDF-1 antibody and found a largely inhibitory effect on neurosphere formation (Figures 2(f)–2(g)). In addition, our ELISA experiment demonstrated presence of local SDF-1 secretion (0.6~0.7 pg/mL) in the culture media. Our results show that the blockage of CXCR4 receptors with a specific antagonist could lead to a decrease in the growth factor-induced progression in NSCs/NPCs.

### 3.3. Blockage of SDF-1/CXCR4 Axis Pathway by AMD Promotes NSCs/NPCs Differentiation In Vitro

**Figure 3:**
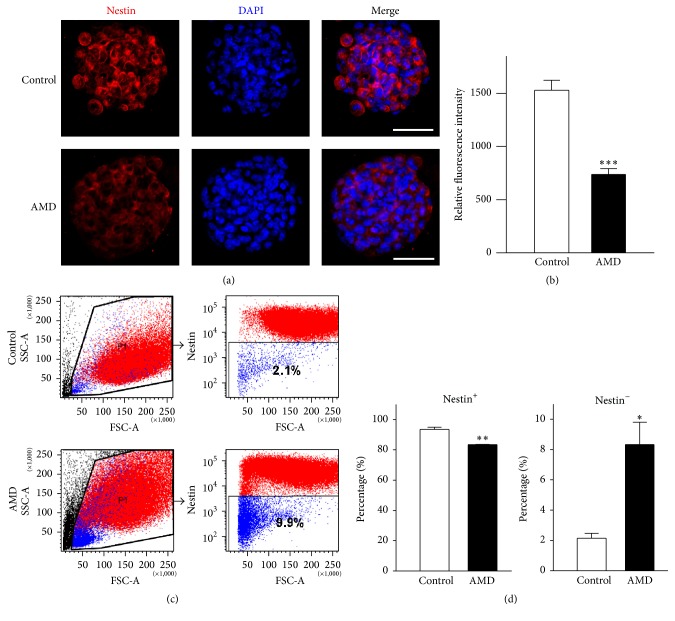
Blockage of CXCR4 signaling transduction impaired the nestin expression in primary neurospheres. (a) Representative images showing nestin (red) in PBS- and AMD-treated neurospheres. DAPI (blue) stained nuclei are used. (b) Quantification of nestin intensity in control or AMD group neurospheres. Data represent the mean ± SEM, control, *n* = 10; AMD, *n* = 11, ^*∗∗∗*^*p* < 0.001. Scale bar, 50 *μ*m. (c) (d) Analysis of nestin^−^ cells in neurospheres after PBS or AMD treatment. Single cell suspensions dissociated from neurospheres were stained and analyzed by flow cytometry. The flow quantification results show a dramatic increase in the percentage of nestin^−^ cells in AMD-treated group. Data are given as mean ± SEM, *n* = 3, ^*∗*^*p* < 0.05, ^*∗∗*^*p* < 0.01, Student's *t*-test.

As mentioned above, depending on the concentration of AMD, application of the dissociated NSCs/NPCs with AMD significantly decreased the number and size of newly formed neurospheres. These results suggest that inhibition of SDF-1/CXCR4 pathway may suppress NSCs/NPCs maintenance in vitro. Neurospheres contain multipotent NSCs, NPCs, and their more differentiated progeny. These heterogeneous spheres can be identified by staining for the intracellular NSCs markers such as nestin, an intermediate filament protein [27]. Thus, to show that the characteristics of neurospheres were influenced by CXCR4 specific antagonists, neurospheres were stained for immunofluorescence using antibodies against nestin for uncommitted NSCs/NPCs. We found high expression of nestin in most of the neurospheres obtained from wild-type mice after 7DIV. However, the fluorescence intensity of the nestin^+^ cells was markedly decreased by the addition of AMD (Figures 3(a)-3(b)), as observed in the 4-OHT-induced gene deletion experiments (Figure 1(d)). To further examine whether SDF-1/CXCR4 signaling played a critical role in the maintenance of NSCs/NPCs characteristics, cells from wild-type primary neurospheres after 7DIV incubation with vehicle or AMD were dissociated and analyzed by flow cytometry technique using a nestin antibody. As shown in Figures 3(c)-3(d), more than 90% of the cells were positive for nestin suggesting that proliferating neurospheres in culture retained their “stemness” properties. Furthermore, our results show that the nestin^+^ cell population was notably decreased after AMD treatment (control, 93.5 ± 1.4%; AMD, 83.3 ± 0.2%, *p* < 0.01). On the contrary, the number of nestin^−^ cells was significantly increased following AMD exposure (control, 2.1 ± 0.3%; AMD, 8.3 ± 1.5%, *p* < 0.05). These results demonstrate that SDF-1/CXCR4 signaling cascades are crucial in NSCs/NPCs maintenance in vitro.

**Figure 4:**
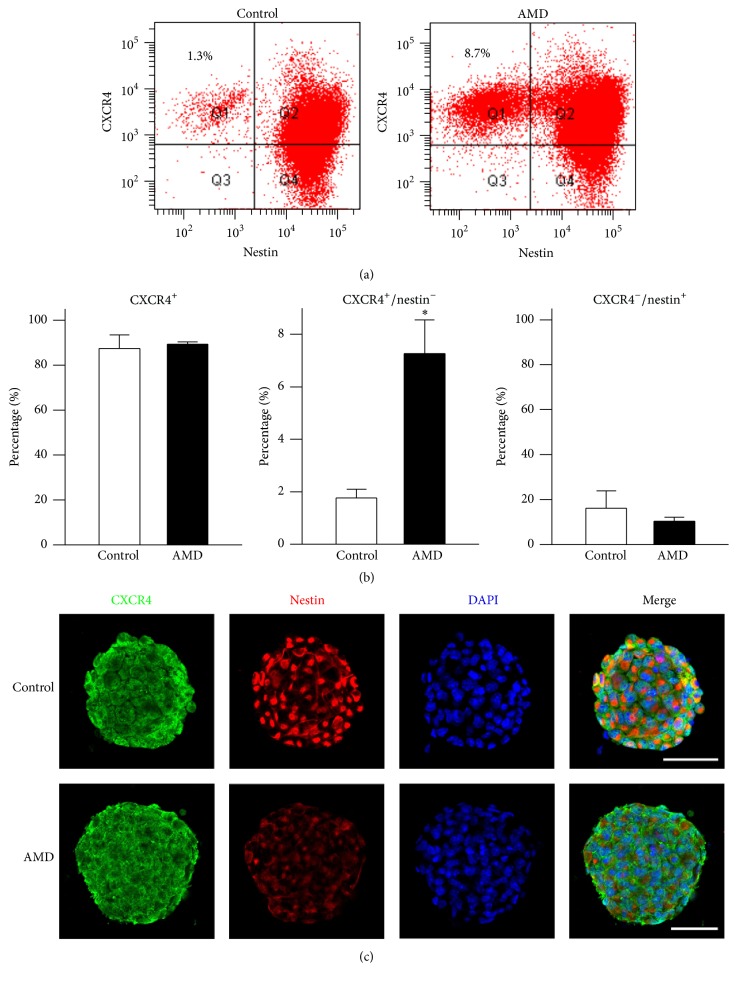
AMD treatment increased the percentage of nestin^−^ cells in CXCR4^+^ population. (a) Primary neurospheres treated with either PBS or AMD were collected for flow cytometric analysis. Numbers in the quadrants indicate the percentage of CXCR4^+^/nestin^−^ cells. (b) Quantification of CXCR4^+^ cells (left panel), CXCR4^+^/nestin^−^ cells (middle panel), and CXCR4^−^/nestin^+^ cells (right panel) in control or AMD-treated group. (c) Representative images showing CXCR4 (green), nestin (red), and DAPI (blue) in PBS- or AMD-treated neurospheres. Results are representative of three independent experiments with neurospheres from three independent litters. Data represent the mean ± SEM, ^*∗*^*p* < 0.05, Student's *t*-test.

In order to specifically address the fact that SDF-1/CXCR4 pathways take part in the NSCs/NPCs maintenance, we next examined the expression profile of nestin in the CXCR4-positive (CXCR4^+^) cells by flow cytometric analysis after AMD application. As shown in Figures 4(a)-4(b), approximately 80% of cells dissociated from primary neurospheres were CXCR4^+^, consistent with previous reports [19, 21]. In addition, there was no significant change on the CXCR4^+^ cells between control and AMD treatment groups. Interestingly, obstruction of CXCR4 signal transduction by AMD markedly increased CXCR4^+^/nestin^−^ cells population (control, 1.8 ± 0.3%; AMD, 7.3 ± 1.3%, *p* < 0.05) implying that SDF-1/CXCR4 cascades may contribute to the sustaining characteristics of NSCs/NPCs. The proportion of CXCR4^−^/nestin^+^ was not significantly altered following AMD application (control, 16.1 ± 7.7%; AMD, 10.4 ± 1.8%, *p* > 0.05). Furthermore, consistent with flow cytometric analysis, we also observed that the immunofluorescence intensity of the nestin^+^ cells was obviously decreased after AMD treatment (Figure 4(c)).

**Figure 5:**
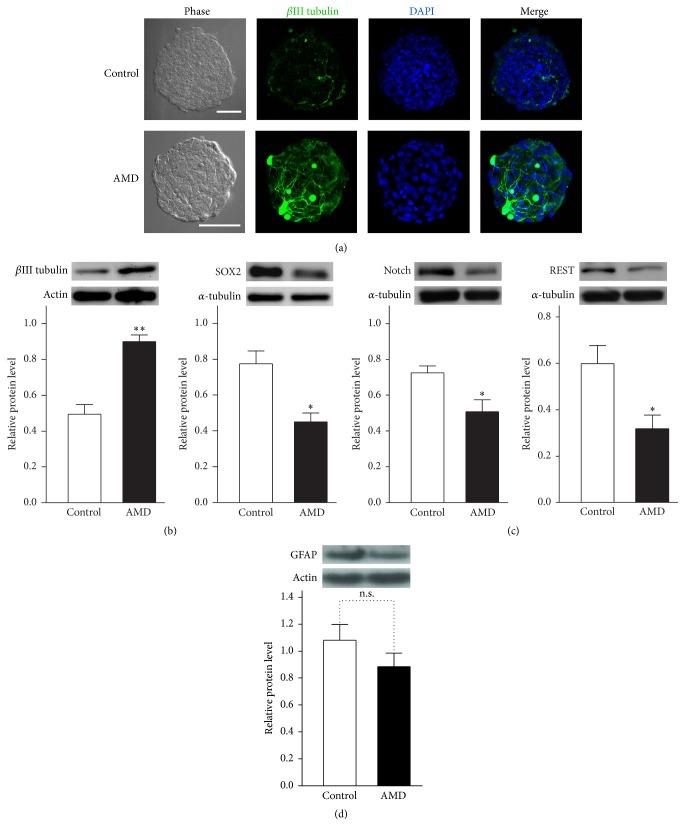
Inhibition of SDF-1/CXCR4 signaling cascades promoted the neuronal lineage differentiation. (a) Confocal microscopic images of *β*III tubulin^+^ (green) cells in the 6DIV neurospheres after PBS or AMD incubation. Scale bar, 50 *μ*m. ((b), (c), and (d)) Western blot of total cell lysates was obtained from mouse primary neurospheres cultures treated with PBS or AMD (1 *μ*g/mL) for 6 days. Antibodies selectively recognizing *β*III tubulin, SOX2, Notch, REST, and GFAP were used. The neurospheres treated with AMD showed a significant increase in *β*III tubulin and Notch. In contrast, the immunodensity exhibited a dramatic downregulation of SOX2 and REST levels after CXCR4 blockage. However, the GFAP protein levels were unchanged after AMD treatment. Results are representative of three independent experiments with neurospheres from three independent litters. Data represent the mean ± SEM, n.s.: no significant, ^*∗*^*p* < 0.05, ^*∗∗*^*p* < 0.01, Student's *t*-test.

Previous studies have shown that elimination of SDF-1/CXCR4 pathways by AMD induced a significant loss of CXCR4-expressing NPCs in the G2/S/M phase through the restrained cell cycle process at the G1 phase [19]. It has been known that the length of G1 phase can directly influence the cell fate determinants of NSCs or NPCs [28]. In addition, several lines of research indicate that prolonging the NPCs G1 phase can promote the cells into a neurogenic-lineage [28–31]. To determine whether blocking CXCR4 with a specific antagonist induced the NSCs/NPCs differentiation toward neuronal fate, we labeled the primary neurospheres with a neuron-specific class III beta-tubulin (*β*III tubulin) antibody [32] to study the distribution of neuronal progeny in the proliferative spheres. Immunocytochemistrical results indicate that the *β*III tubulin immunoreactivity was remarkably enhanced after 7 days of AMD incubation (Figure 5(a)). Moreover, our immunoblotting analysis reveals that the *β*III tubulin level was strongly elevated in 7DIV NSCs/NPCs culture in the presence of AMD. In contrast, the SOX2 protein expression has been found at a lower level (Figure 5(b)). Remarkably, the quantities of Notch or REST, which, respectively, play an essential role in NSCs/NPCs maintenance and neuronal differentiation [33–35], was found to be downregulated in the AMD treatment groups (Figure 5(c)). The NSCs/NPCs have been shown to be able to produce nonneuronal cells (such as astrocyte). We therefore asked whether the AMD treatment can influence the glial lineage cells derived from NSCs/NPCs. However, we did not observe a significant difference in glial fibrillary acidic protein (GFAP, a specific astrocyte marker) levels between control-treated and AMD-treated group (Figure 5(d)).

**Figure 6:**
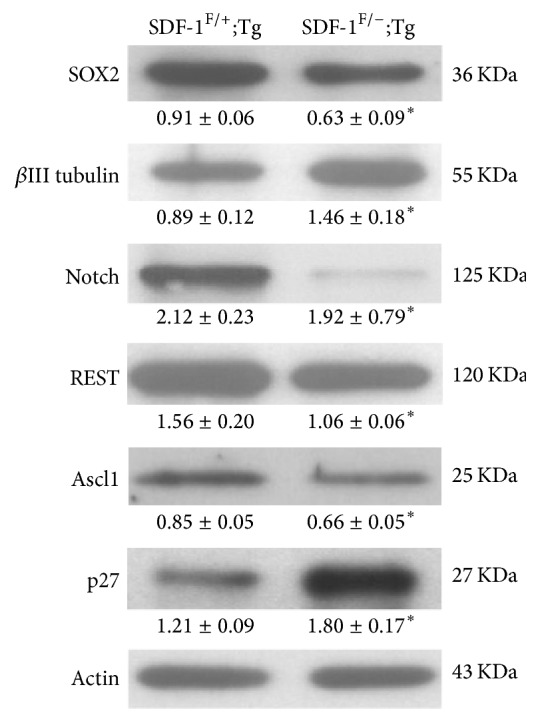
Loss of SDF-1/CXCR4 signaling pathway promotes neural fate commitment via activation of neural lineage genes. Whole cell lysates were prepared from SDF-1^F/+^;Tg or SDF-1^F/−^;Tg group after 4-OHT treatment. Western blot analysis was conducted using anti-SOX2, *β*III tubulin, Notch, REST, Ascl1, and p27 antibodies. The numbers under each blot are intensity of the blot relative to that of internal control. Results are representative of three independent experiments with neurospheres from three independent litters. Data represent the mean ± SEM, ^*∗*^*p* < 0.05, Student's *t*-test.

To further confirm the above observations, we also used the conditional gene deletion strategy to measure the potential changes of the NSCs/NPCs compartment by western blotting after SDF-1 elimination. In parallel with the AMD-treated results, our results show that the SOX2 level was significantly decreased following the SDF-1 deletion; otherwise the *β*III tubulin expression was strongly elevated. Moreover, we also observed a dramatic decline in the Notch and REST immunodensity after 4-OHT-induced gene deletion of SDF-1 in primary neurospheres culture. Unexpectedly, we observed a dramatic decrease in Ascl1 level, a proneural gene known to promote cell cycle exit and neuronal differentiation in diverse NSCs/NPCs populations [36]. This suggests that SDF-1 may play an unreported role in Ascl1 regulation. Interestingly, we found that the antiproliferative gene p27, a cyclin-dependent kinase inhibitor which has been known to delay G1/S transition and promote neuronal differentiation in the mouse CNS [37, 38], was significantly increased in SDF-1^F/−^;Tg group (Figure 6). Our results suggest that there is a requirement for SDF-1/CXCR4 signal transduction in maintenance of NSCs/NPCs. Inactivation of SDF-1/CXCR4 signal pathway may decrease its self-renewal ability and leads to premature neuronal differentiation.

### 3.4. CXCR4 Blockage during the Proliferation Period Affects Subsequent Neuronal Differentiation of NSCs/NPCs

**Figure 7:**
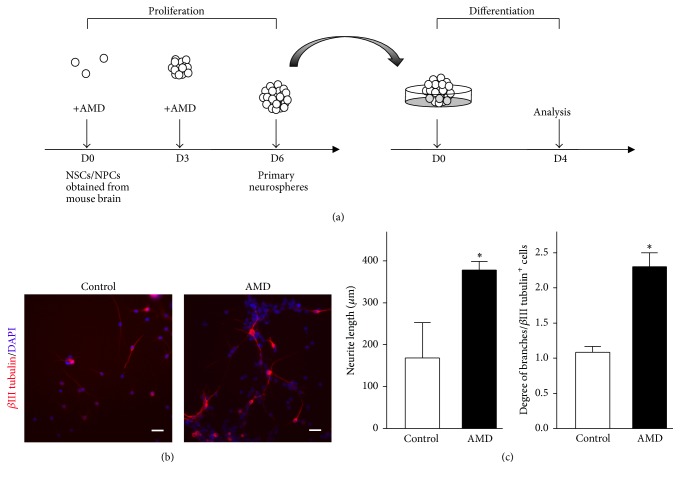
Blocking of CXCR4 transduction facilitated the neurites outgrowth and branching in differentiated neurons. (a) Schematic representation of the protocol used in this study. (b) Representative images of *β*III tubulin^+^ (red) cells cultured from mouse brain-derived neurospheres after 6DIV PBS or AMD treatment. The neurospheres were reseeded onto a poly-D-lysine-coated dish and allowed to differentiate for 4 days. Scale bar, 25 *μ*m. (c) Average neurite length (left panel) and branching degree (right panel) of *β*III tubulin^+^ cells, where the AMD-treated group showed significant increase in both length and branching level. Data represent the mean ± SEM, control, *n* = 3; AMD, *n* = 4, ^*∗*^*p* < 0.05, Student's *t*-test.

Our results reveal that inhibition of SDF-1/CXCR4 axis pathway increases the proportion of neuronal progeny in the neurospheres. This phenomenon leads us to examine whether SDF-1/CXCR4 signaling cascade blockage would induce premature neuronal differentiation of NSCs/NPCs. Primary NSCs/NPCs were growing at AMD medium with bFGF and EGF. After 6 days of incubation, neurospheres were collected and then reseeded onto a poly-D-lysine coated dish in the mitogen-free medium with 1% FBS for differentiation (Figure 7(a)). To address the neuronal maturation, we next quantified the length and number of branch points of neurites in differentiating neurons labeled with *β*III tubulin antibody. We found that both length and branching of neuritis were significantly increased in AMD-exposed group (Figures 7(b)-7(c)). Our results suggest that AMD-treated proliferating NSCs/NPCs exhibit subsequent differentiation associated increases in neuronal maturation.

## 4. Discussion

In the present study, we demonstrated that CXCR4 receptor is highly expressed in mouse NSCs/NPCs and responsive to its primary ligands—SDF-1, which is secreted by cultured neurospheres. Obstruction of SDF-1/CXCR4 signaling transduction impairs the maintenance of self-renewal ability of NSCs/NPCs which results in exhaustion of their stemness characteristics. Moreover, we have shown that either blockage of CXCR4 by specific inhibitors, AMD, or conditional deletion of SDF-1 in neurospheres will enhance the expression of their neuronal progenies accompanied with Notch, REST, Ascl1, and p27 level change. Our results suggest that loss of functional SDF-1/CXCR4 signaling pathway in NSCs/NPCs induces cell cycle exit and promotes premature neural differentiation.

Neurogenesis is a dynamic process of generating functional neurons from NSCs/NPCs in the mammalian nervous system. These phenomena are known to be regulated by numerous cell-intrinsic transcription factors or extrinsic microenvironment niches to govern self-renewal or differentiation [1, 39]. Recently, evidence suggests that the chemokines, small molecules responsible for the diverse functions of cells in the immune system, and their receptors may play an important role in normal neurogenesis [10]. In particular, previous studies have demonstrated that SDF-1 and CXCR4 not only are highly enriched throughout the developing and adult nervous system but also have an essential role for the normal brain architecture [40, 41]. Genetic deletion of the gene for SDF-1 or CXCR4 results in embryonic lethality accompanied with a dramatic alterations in the development of the cerebellum, cortex, and hippocampus due to abnormal positioning of neuronal precursor cells [14, 15, 42]. In the current study, we have identified a high level of CXCR4 expression on nestin^+^ or SOX2^+^ NSCs/NPCs obtained from postnatal day 1 mouse brain. In addition, our in vitro studies reveal that these NSCs/NPCs retain their multipotency and are able to differentiate to astrocytes, neuron, and oligodendrocytes. Interestingly, blockage of SDF-1/CXCR4 signal transduction by either AMD or genetic deletion of SDF-1 significantly decreases neurospheres formation. This phenomenon contributes to downregulation of the cells' self-renewal ability. Consistent with these observations, previous reports suggest that inhibition of SDF-1/CXCR4 axis by AMD in embryonic NPCs culture (derived from CXCR4-GFP transgenic mice) dramatically decreases the spheres-forming capacity [19]. Furthermore, we found that application of specific antibody against SDF-1 in culture medium also significantly obstructs the neurospheres outgrowth which implies that SDF-1 was secreted by NSCs/NPCs locally. Indeed, our ELISA assays support this notion as abundant SDF-1 were found in the neurospheres culture media. This is consistent with previous studies that also detected SDF-1 in the conditioned medium in mouse embryonic NPCs cultures [19]. Neurons and astrocytes are known to be the source of SDF-1 production both in vivo and in vitro [40, 43] and play a novel role in adult neurogenic processes [40]. Therefore, NSCs/NPCs may also produce and release SDF-1 by themselves in response to normal neurogenesis.

Previous studies indicate that CXCR4^+^-NPCs were more vulnerable to AMD treatment [19]. Downregulation of proliferative ability in NSCs/NPCs caused by AMD or SDF-1 knockout may be a consequence of increased cells quiescence or cell lineage differentiation, although there are no direct evidences that report the effect of SDF-1 on regulation of NSCs/NPCs differentiation. It has been demonstrated that SDF-1 coexists in GABA-containing vesicles in the terminals of basket cells, regulates the strength of GABAergic input to nestin^+^-type 2 NPCs in hippocampal DG, and plays a crucial role in adult neurogenesis [40]. GABA is one of the major extrinsic factors that promotes neuronal differentiation in adult hippocampal type 2 NPCs [44]. Therefore, SDF-1 is likely to be a regulator in NPCs maintenance and differentiation. However, the mechanisms between GABA and SDF-1 on NPCs differential regulation require further investigations. Interestingly, our experiments indicate that elimination of SDF-1/CXCR4 signal pathway caused a predominant reduction in nestin fluorescence intensity and nestin^**+**^ cell populations in cultured neurospheres. Moreover, we observed that the CXCR4^+^-nestin^−^ cells population was dramatically increased after AMD application. These results suggest that SDF-1/CXCR4 transduction might play an important role in maintenance of NSCs/NPCs stemness. To further investigate the physiological functions of SDF-1 in neurogenesis, we also analyzed the in vivo activity of NSCs/NPCs in DG following SDF-1 gene deletion. Similarly, we found a clearly downward trend in the proliferative NSCs/NPCs in dentate region. However, we did not observe any significant differences in DCX-positive cells pattern. These results suggest that some compensatory mechanisms may exist to reverse the SDF-1 deficiency in vivo. Although the detailed mechanism of SDF-1/CXCR4 signaling in the preservation of NSCs/NPCs characteristics is still unknown, it has been demonstrated that SDF-1/CXCR4 signaling is functionally crucial for maintenance of stemness and fate decisions in mouse spermatogonial stem cells. Ablation of SDF-1-CXCR4 cascade in undifferentiated spermatogonia results in spermatogonial stem cells loss and generates more differentiated progeny after retinoic acid stimulation [45].

Interestingly, we observed the neuronal progeny was significantly elevated following 4-OHT-induced deletion of SDF-1 or AMD treatment. Moreover, we also found that either Notch or REST remarkably declined after CXCR4 blockage. These findings indicate that SDF-1/CXCR4 pathway might govern the Notch and REST expression during the neurogenesis. Notch and REST are known to play important roles in NSCs/NPCs maintenance and neuronal differentiation, respectively. Genetic deletion of Notch or REST signaling caused NSCs to be prematurely differentiated into neurons and NSCs pool exhaustion [33, 34, 46]. Although we do not know the mechanism of how the SDF-1/CXCR4 cascade regulates Notch or REST levels in NSCs/NPCs previous reports revealed that SDF-1 can directly stimulate Wnt/beta-catenin transcriptional activity in rat NPCs [47]. Elimination of SDF-1/CXCR4 signal transduction may cause downregulation of Wnt/beta-catenin cascades. Wnt/beta-catenin signaling is a hallmark of self-renewing activity in vivo, blockage of Wnt/beta-catenin pathway has been found to promote neuronal induction in mouse embryonic stem cells [48]. Moreover, Wnt/beta-catenin signaling has been demonstrated to regulate Notch1 or REST expression in hematopoietic stem cells and chick spinal cord neural precursor cells [49, 50]. Further studies will be needed to elucidate the potential role of SDF-1/CXCR4 pathway in Notch or REST gene modulation.

Association of SDF-1 with CXCR4 activates multiple signaling pathways. For instance, SDF-1 can promote human NPCs proliferation by increasing Akt/FOXO3a signaling [51]. FOXO3a is an important modulator in cell cycle progression at G1/S transition by controlling the cyclin-dependent kinase inhibitor p27 transcription [52]. Overexpression of FOXO3a suppresses endothelial progenitor cells proliferation and p27 upregulation and caused a noticeable increase in G1 frequencies. While we did not provide FOXO3a activity in our 4-OHT-induced SDF-1 knockout experiment, we note that the p27 was significantly increased following SDF-1 gene deletion. Therefore, the increment of p27 level may contribute to modulation of subsequent G1/S transition. The length of G1 phase has been shown to determine the cellular fate of NSCs or NPCs [28]. Extensive evidence revealed that prolonging the NPCs G1 phase can induce the cells into a neurogenic-lineage [28–31]. Whether elimination of SDF-1/CXCR4 pathway is required for prolonged G1 phase mediated by FOXO3a/p27 in NSCs/NPCs remains unclear. Genetic deletion of the CXCR4 receptors results in abnormal cell cycle transition from G0-G1 in hematopoietic stem cells [53]. Furthermore, previous reports have demonstrated that CXCR4-specific antagonist impairs the bFGF/EGF-induced self-renewal ability in embryonic NPCs due to arrest of cell cycling at the G1 phase [19].

As mentioned above, we found that loss of SDF-1/CXCR4 axis causes enhanced neuronal differentiation of the mouse NSCs/NPCs. Unexpectedly, our in vitro study observed a dramatically reduced Ascl1 expression, a proneural gene, which is known to promote cell cycle exit and neuronal differentiation during the adult hippocampal neurogenesis [36, 46]. Ascl1 level was stage-specific in granule neuron development in hippocampal DG and dynamic with respect to REST level [34]. For instance, Ascl1 was highly expressed in type 2a and 2b NSCs but not type 1 and type 3 NSCs or immature/mature neuron. In this study, we observed that both nestin-expressed cells or SOX2 transcripts were significantly decreased after SDF-1/CXCR4 signaling elimination, whereas the immature neuron marker (*β*III tubulin) was dramatically increased. This result suggests that SDF-1/CXCR4 signaling may play an unreported role in Ascl1 regulation. Further studies are needed to elucidate the association between SDF-1-mediated signaling and Ascl1. Taken together, our findings indicate the SDF-1/CXCR4 signal axis may play a role in the regulation of proneuronal gene to prevent precocious neuronal differentiation. Indeed, our in vitro AMD blockage results suggest that suppression of CXCR4 signaling during the sphere forming period induces a premature development in the subsequent differentiation of NSCs/NPCs.

## 5. Conclusions

Our study suggests a potential role of SDF-1/CXCR4 signaling in the maintenance of NSCs/NPCs characteristics. Loss of SDF-1 or CXCR4 repression causes a precocious differentiation of neuronal precursor cells resulting from abnormal change in Notch, REST, Ascl1, and p27 signaling. Further work is required to understand the related genes and signaling pathways via regulation of SDF-1/CXCR4 signaling controlling the NSCs/NPCs behaviours.

## Supplementary Material

This supplementary material contains evidence of: (1) Characteristics of cultured neurospheres. (2) Loss of SDF-1 transcripts in neurosphere cultures after 4-OHT-treatment. (3) (4) Effect of SDF-1 gene deletion on mouse hippocampal neurogenesis.
