# Supplementary material for: SDF-1/CXCR4 Signaling Maintains Stemness Signature in Mouse Neural Stem/Progenitor Cells
Source: Stem Cells Int. 2017 Mar 20;2017:2493752. doi: 10.1155/2017/2493752 (PMC5376953; doi:10.1155/2017/2493752)
Supplement: Supplementary file 1 — This supplementary material contains evidence of: (1) Characteristics of cultured neurospheres. (2) Loss of SDF-1 transcripts in neurosphere cultures after 4-OHT-treatment. (3) (4) Effect of SDF-1 gene deletion on mouse hippocampal neurogenesis. [file 2493752.f1.pdf]

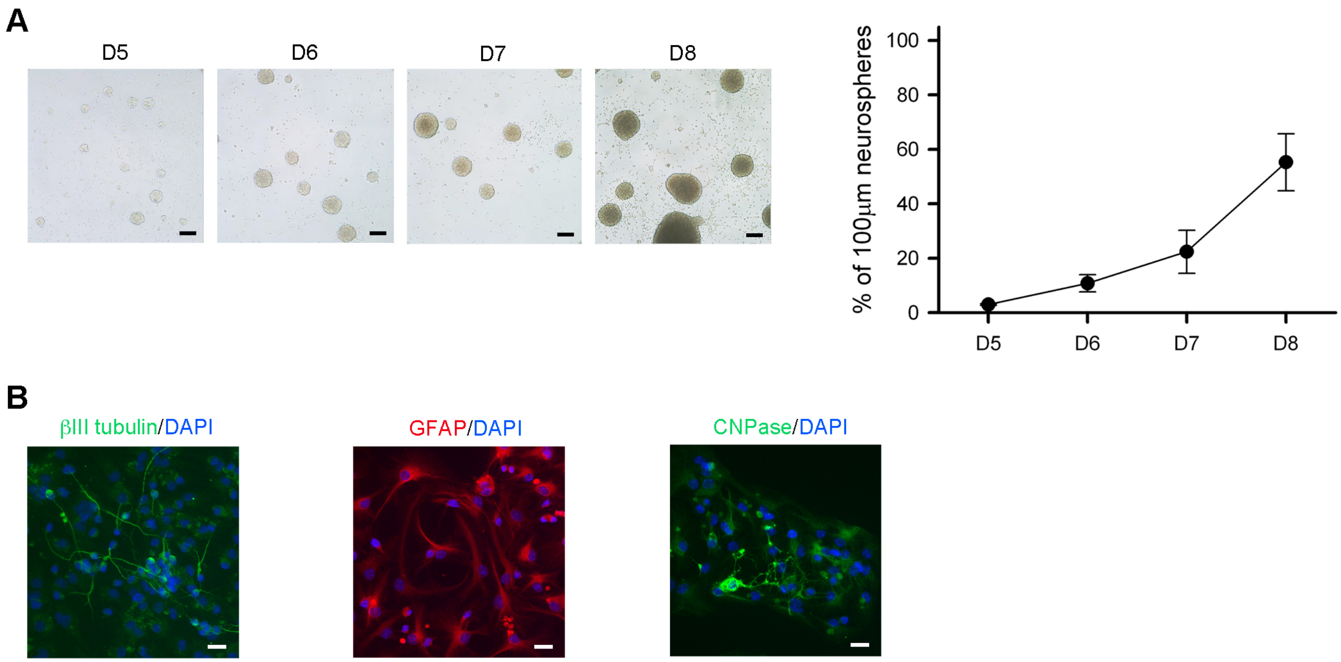
A
D5
D6
D7
D8
% of 100μm neurospheres
100
80
60
40
20
0
D5
D6
D7
D8
B
βIII tubulin/DAPI
GFAP/DAPI
CNPase/DAPI

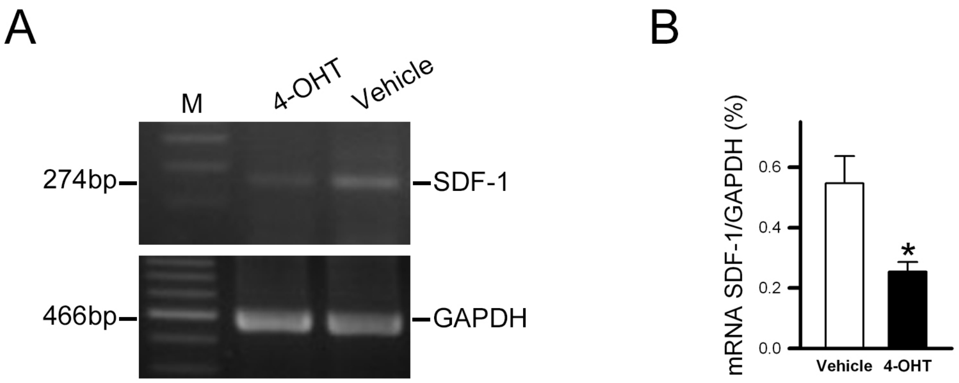
A
M
4-OHT
Vehicle
274bp
SDF-1
466bp
GAPDH
B
mRNA SDF-1/GAPDH (%)
0.6
0.4
0.2
0.0
*
Vehicle
4-OHT

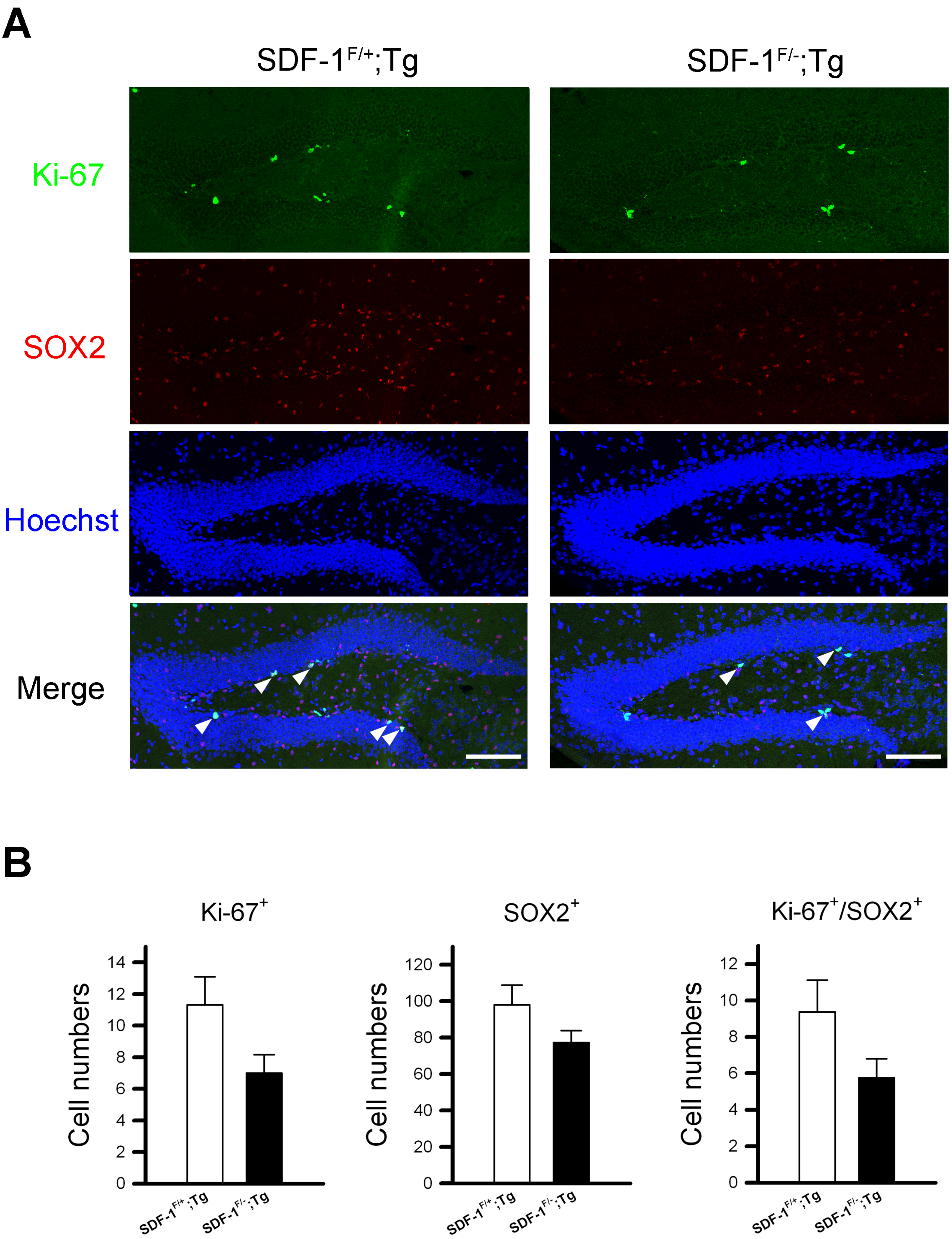
A
SDF-1F/+;Tg
SDF-1F/-;Tg
Ki-67
SOX2
Hoechst
Merge
B
Ki-67+
SOX2+
Ki-67+/SOX2+
Cell numbers
0
2
4
6
8
10
12
14
20
40
60
80
100
120
SDF-1F/+;Tg
SDF-1F/-;Tg

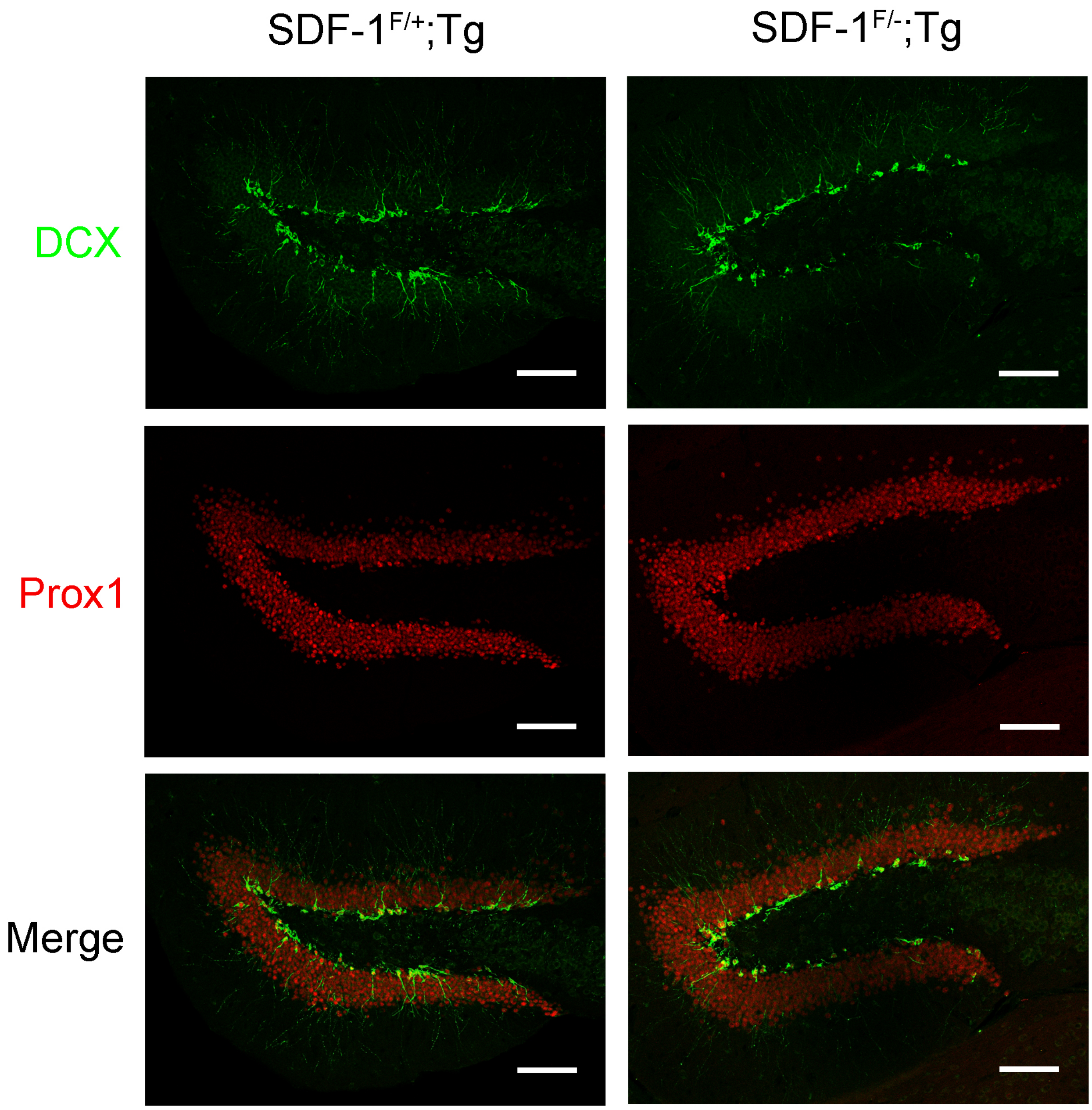
SDF-1F/+;Tg
SDF-1F/-;Tg
DCX
Prox1
Merge

Supplemental Figure Legends

S1. Primary neurospheres culture derived from postnatal mouse brains expressed NSCs/NPCs characteristics.

Primary NSCs/NPCs obtained from postnatal day 1 mice were cultured in a density of 2×10^5 cells/ml. (A) Representative images of neurospheres from 5DIV to 8DIV. The neurospheres showed a significant increase in size during the cultured period (left panel). Scale bar, 100 μm. Percentage of neurospheres with large size (>100μm) among the total number of neurospheres. Error bars represent S.E.M. of the mean. (right panel) (B) Cells dissociated from neurospheres were multipotent and able to differentiate to neurons (βIII tubulin), astrocytes (GFAP) and oligodendrocytes (CNPase). Scale bar, 20 μm.

S2. Loss of SDF-1 transcripts in neurosphere cultures after 4-OHT-treatment.

Neurospheres obtained from SDF-$1^{F/-}$;Tg mice were treated with vehicle or with 4-OHT and then harvested on 6DIV. (A) RT-PCR assays performed on total cellular RNA demonstrated significantly decreased levels of SDF-1 transcripts after 4-OHT treatment. (B) Comparison of the relative SDF-1 mRNA levels between two groups of samples after 4-OHT treatment. Data represent the mean ± S.E.M., * $p<0.05$, n=2 each, Student’s *t*-test.

S3. Deletion of SDF-1 gene showed a decreasing trend in proliferation of NSCs/NPCs in hippocampal DG.

(A) Four weeks SDF-1$^{F/+}$;Tg or SDF-1$^{F/-}$;Tg mice were received 5 doses of tamoxifen (TAM) in 1-days intervals. Brain samples were collected 1 month after the last TAM injection. Ki-67 (green) or SOX2 (red) were used to label proliferative cells and NSCs/NPCs, respectively, after TAM-induced gene deletion. SDF-1$^{F/-}$;Tg mice exhibited a downward trend in proliferation of NSCs/NPCs in the DG as compared to SDF-1$^{F/+}$;Tg littermates. Arrows head indicate the Ki-67^{+}/SOX2^{+} cells in or adjacent to the subgranular zone in dentate area. Scale bar, 100 μm. (B) Quantification of Ki-67^{+}, SOX2^{+}, and Ki-67^{+}/SOX2^{+} cells in DG. Data represent the mean ± S.E.M., n=2 different mice and 16 total sections per group (Ki-67^{+}, p=0.053, SOX2^{+}, p=0.114, Ki-67^{+}/SOX2^{+}, p=0.084, Student's t-test).

S4. Conditional deletion of SDF-1 gene in adolescent mice did not alter the DCX expression in hippocampus.

Representative images of DCX (green) and a dentate granule cell specific marker, Prox1 (red), expression in SDF-1$^{F/-}$;Tg and SDF-1$^{F/+}$;Tg mice after 1 month of TAM injection. There were

no significant differences in DCX level between two groups in DG. Scale bar, 100 μm.
